# Trajectories of Brain Lactate and Re-visited Oxygen-Glucose Index Calculations Do Not Support Elevated Non-oxidative Metabolism of Glucose Across Childhood

**DOI:** 10.3389/fnins.2018.00631

**Published:** 2018-09-11

**Authors:** Helene Benveniste, Gerald Dienel, Zvi Jacob, Hedok Lee, Rany Makaryus, Albert Gjedde, Fahmeed Hyder, Douglas L. Rothman

**Affiliations:** ^1^Department of Anesthesiology, Yale School of Medicine, Yale University, New Haven, CT, United States; ^2^Department of Neurology, University of Arkansas for Medical Sciences, Little Rock, AR, United States; ^3^Department of Cell Biology and Physiology, University of New Mexico, Albuquerque, NM, United States; ^4^Department of Anesthesiology, Stony Brook University, Stony Brook, NY, United States; ^5^Department of Translational Neurobiology, University of Southern Denmark, Odense, Denmark; ^6^Department of Biomedical Engineering & Radiology and Biomedical Imaging, Yale School of Medicine, Yale University, New Haven, CT, United States

**Keywords:** non-oxidative metabolism, aerobic glycolysis, brain, child, development, lactate, bioenergetics

## Abstract

Brain growth across childhood is a dynamic process associated with specific energy requirements. A disproportionately higher rate of glucose utilization (CMR_glucose_) compared with oxygen consumption (CMR_O2_) was documented in children's brain and suggestive of non-oxidative metabolism of glucose. Several candidate metabolic pathways may explain the CMR_glucose_-CMR_O2_ mismatch, and lactate production is considered a major contender. The ~33% excess CMR_glucose_ equals 0.18 μmol glucose/g/min and predicts lactate release of 0.36 μmol/g/min. To validate such scenario, we measured the brain lactate concentration ([Lac]) in 65 children to determine if indeed lactate accumulates and is high enough to (1) account for the glucose consumed in excess of oxygen and (2) support a high rate of lactate efflux from the young brain. Across childhood, brain [Lac] was lower than predicted, and below the range for adult brain. In addition, we re-calculated the CMR_glucose_-CMR_O2_ mismatch itself by using updated lumped constant values. The calculated cerebral metabolic rate of lactate indicated a net influx of 0.04 μmol/g/min, or in terms of CMR_glucose_, of 0.02 μmol glucose/g/min. Accumulation of [Lac] and calculated efflux of lactate from brain are not consistent with the increase in non-oxidative metabolism of glucose. In addition, the value for the lumped constant for [^18^F]fluorodeoxyglucose has a high impact on calculated CMR_glucose_ and use of updated values alters or eliminates the CMR_glucose_-CMR_O2_ mismatch in developing brain. We conclude that the presently-accepted notion of non-oxidative metabolism of glucose during childhood must be revisited and deserves further investigations.

## Introduction

Understanding the metabolic needs of the developing brain is essential for maintaining brain health across childhood and during adolescence. Information on the bioenergetic state of normal children's brain during development remains limited due to ethical concerns and overall complexity of conducting quantitative cerebral metabolic studies using positron emission tomography (PET) or magnetic resonance spectroscopy (MRS). Filling this gap in knowledge may shed light on several clinical predicaments and disease states including understanding the high incidence of benign febrile seizures in children 18 month of age (Pavlidou et al., 2013), the increased risk of long-term cognitive sequelae from multiple anesthesia and surgeries exposures before age 4 years (Glatz et al., 2017), and the higher rate of brain overgrowth observed in children with autism spectrum disorder (Hazlett et al., 2005; Sacco et al., 2015).

In humans, brain growth is rapid after birth and the young brain reaches adult-sized volume around age six (Giedd et al., 1999; Lenroot and Giedd, 2006; Semple et al., 2013). Neuronal maturational processes and myelination rates are dynamic and varying across the cortex (Bauernfeind and Babbitt, 2014). Synaptic density peaks at 2–3 years of age followed by pruning and decreased number (Huttenlocher, 1990; Semple et al., 2013). Myelination rate remains high until age 10 years (Miller et al., 2012). The growth pattern of brain development is paralleled by age-varying energy requirements.

The brain relies predominantly on glucose for energy, and PET is used to measure rates of glucose consumption (CMR_glucose_) with the glucose analog [^18^F]fluorodeoxyglucose (FDG) and oxygen consumption (CMR_O2_) with ^15^O-O_2_ (Raichle et al., 1976; Mintun et al., 1984; Reivich et al., 1985; Ohta et al., 1992; Gjedde and Marrett, 2001), allowing for calculation of the oxygen-glucose index (OGI = CMR_O2_/CMR_glucose_). The theoretical maximum for OGI is 6.0 (6O_2_ + 1 glucose → 6CO_2_ + 6H_2_O) when no other substrates are utilized, and the OGI therefore falls below 6 when glucose is consumed but not oxidized. The disproportionate utilization of glucose compared with oxygen *in the presence of normal oxygen delivery* is a phenomenon often called “aerobic glycolysis” in the literature (Hertz et al., 1998; Vaishnavi et al., 2010; Goyal et al., 2014; Dienel and Cruz, 2016; Hyder et al., 2016). However, to avoid confusion, since glycolysis can be upregulated under either aerobic or hypoxic/anaerobic conditions, we refer here to non-oxidative metabolism of glucose as glycolytic production of lactate that is not oxidized and/or of utilization of glucose by any other pathways that do not consume oxygen via the mitochondrial electron transport chain (e.g., glycogen synthesis, pentose phosphate shunt activity, biosynthetic reactions, etc.).

Chugani et al. reported that cortical CMR_glucose_ in newborns was ~20–35% lower than in adults, and increased rapidly over the first 1–3 years (Chugani et al., 1987). In 3–8 year old children, CMR_glucose_ was twice adult values, followed by a gradual decrease from 4 to 15 years to attain lower adult levels (Chugani et al., 1987). These values have become widely accepted and form the basis of proposals regarding metabolic adaptations in the developing human brain. Goyal et al. (2014) recently extended these findings by performing a meta-analysis based on the data from Chugani et al. and other studies to map trajectories of CMR_glucose_ and CMR_O2_, across the human lifespan and reported a 33% peak of excess CMR_glucose_ over CMR_O2_ at 3–5 years of age (Goyal et al., 2014) and an OGI of ~4.1, inferring enhanced non-oxidative metabolism of glucose during early childhood (Goyal et al., 2014). By analogy to cancer cell growth—where an elevated non-oxidative metabolism of glucose is thought to support accelerated uptake and incorporation of nutrients into the growing cancer biomass (Vander Heiden et al., 2009)—it has been proposed that an elevated non-oxidative metabolism of glucose in the developing brain would support growth, axonal elongation synaptogenesis, and remodeling (Bauernfeind et al., 2014; Goyal et al., 2014).

**Figure 1:**
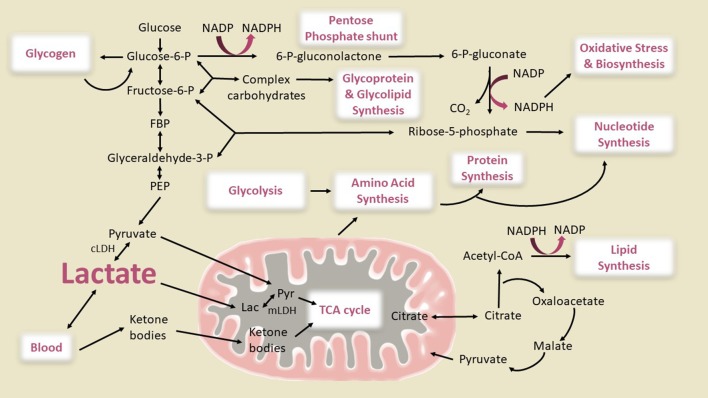
Metabolic pathways of importance for the developing brain. Glycolysis, oxidative phosphorylation via the citric acid (TCA) cycle and the pentose phosphate pathway generating NADPH, and the use of ketone bodies as supplemental fuel are shown. The connections between glycolysis, complex carbohydrate, amino acid, protein, lipid, and nucleotide synthesis are also illustrated. The pathway fluxes that change during brain development to cause glucose utilization in excess of oxygen (enhanced non-oxidative metabolism of glucose) are not known. Glucose can be converted to lactate directly via the glycolytic pathway or after shunting through glycogen or the pentose shunt pathway, then either oxidized in the mitochondria or released from brain. Our diagram shows two pathways for mitochondrial lactate oxidation, direct lactate transport into the mitochondria and oxidation as has been reported in studies of muscle and brain (Brooks, 1986, 2000, 2018; Schurr, 2006; Passarella et al., 2014; Rogatzki et al., 2015) and conversion of lactate to pyruvate in the cytosol by cytosolic lactate dehydrogenase (cLDH) and subsequent transport into inner matrix of the mitochondria through pyruvate transporters. Although in muscle it has been reported that the large majority of lactate is directly oxidized in the mitochondria by mitochondrial LDH (mLDH), the effective blocking of glucose oxidation in brain cell cultures, synaptosomes, and brain slices by inhibition of the malate aspartate shuttle (MAS) (which transports the redox equivalent from NADH produced by cLDH into the mitochondria) (Fitzpatrick et al., 1983; Kauppinen et al., 1987; Cheeseman and Clark, 1988; Mckenna et al., 1993) and the inability of L-lactate to rescue glutamate toxicity in MAS-knockout neurons whereas it does in wild type (Llorente-Folch et al., 2016) suggests that brain mitochondria mainly use cytosolic pyruvate as an oxidative source. Also, LDH is considered to be a cytoplasmic marker in subcellular fractionation studies of brain (Johnson and Whittaker, 1963; Tamir et al., 1972), and <1% of the LDH in a brain homogenate is recovered in purified mitochondria (Lai and Clark, 1976; Lai et al., 1977). However, from the standpoint of this study the two pathways of lactate oxidation would lead to the same OGI as shown in Figure 2. Glucose can also be used for synthesis of glycogen, amino acids, proteins, complex carbohydrates, lipids, glycolipids, and glycoproteins, and nucleotides. The flux of the pentose shunt in developing brain is higher than in adult brain even though maximal capacity is similar at all ages (Baquer et al., 1977). The illustration is based metabolic pathways active in proliferative cells to explain the Warburg effect that involves aerobic glycolysis and lactate efflux (Vander Heiden et al., 2009). Warburg theories for cancer cells state that the increased glucose uptake is shunted through the pentose phosphate pathway for the additional NADPH needed for biosynthetic reactions. Theoretically, 5/6 of the glucose entering the oxidative branch of the pentose phosphate pathway should end up as lactate and be exported from the brain. However, if recycling of Fru-6-P back into the pentose shunt is complete, this pathway can contribute a higher fraction to the consumption of glucose in excess of oxygen (see text). It is an open question how much NADPH is needed to meet the biosynthetic needs for synaptogenesis. CoA, Coenzyme A; P, phosphate; FBP, fructose-1,6-P_2_; PEP, phosphoenolpyruvate. Modified from Figure 3 of Vander Heiden et al. (2009) with permission of the authors. Reprinted with permission from AAAS.

However, conversion of all of the glucose consumed in excess of oxygen into brain biomass would cause an impossibly large increase in brain size, doubling within a month. It is necessary, therefore, to search for potential explanations for the large magnitudes of non-oxidative metabolism of glucose reported by Goyal et al. (2014), which is several-fold higher than in the adult brain (Hyder et al., 2016). Although a lower than normal OGI in children's brain is suggestive of increased glycolytic flux or non-oxidative metabolism of glucose, the downstream fate of the glucose carbon has not been established. In other words, the “OGI” by itself provides no information about the fate of excess glucose utilization which can involve many pathways as shown in Figure 1.

A possibility is that a higher-than-normal lactate production may explain the elevated non-oxidative metabolism of glucose reported in the developing brain (Goyal et al., 2014). To assess this concept, we measured steady state lactate concentrations ([Lac]) from brains of 87 children who underwent routine MRI examination under anesthesia (Jacob et al., 2012) using proton magnetic resonance spectroscopy (^1^H MRS), and found that lactate accumulation and efflux could not explain the excess non-oxidative utilization of glucose. We, therefore, also evaluated several other possibilities including the storage of the excess glucose uptake into glycogen, and its complete oxidation or shunting away from lactate via the pentose phosphate pathway. At present there is no strong evidence for these possibilities although they will need to be directly measured before definite conclusions can be made. We then examined the alternate possibilities of age dependence on the conversion of the FDG PET measurement into a calculated rate of CMR_glucose_ and the impact of plasma ketones, lactate, and other non-glucose substrates on the OGI calculation. We found that the “lumped constant,” which is the constant used for the conversion of FDG phosphorylation to CMR_glucose_, of Chugani et al. (1987) was considerably lower than modern accepted values and tested the impact of updated values on OGI.

Based on our direct measurements of brain [Lac] and calculations as well as mass balance considerations, we conclude that the claims of net lactate efflux and/or conversion of glucose into brain mass explaining the enhanced non-oxidative metabolism of glucose in children compared to adults are incorrect. There are several other potential metabolic sources of the reported glucose uptake/oxidation mismatch, including alternate pathways of glucose metabolism or even other substrates. For example, ketone metabolism is known to be higher in children, but this would reduce the measured rate of non-oxidative metabolism of glucose by increasing the OGI. However, there are limited measurements available on these alternate pathways. We show through simulations that a more likely explanation, at present, is the use of different lump constants in PET CMR_glucose_ data in children and adults. When modern LC values are used with the originally reported Chugani et al. results (Chugani et al., 1987), the difference in non-oxidative metabolism of glucose between adults and children disappears. However, until the necessary studies are performed, our present understanding of glucose metabolism in the developing brain, despite being widely accepted, is at best incomplete and potentially largely incorrect and deserves further investigation. The non-oxidative metabolism of glucose “story” is more complex than conversion of glucose into brain mass or lactate.

## Materials and methods

De-identified ^1^H MRS spectra and anatomical T1-weighted scans from 87 children (age: 2–7 years, 38 females and 49 males) undergoing diagnostic MRI under anesthesia were included in the analysis. ^1^H MRS metabolite data from 60 of the 87 children included were previously reported in a study not focused on lactate but documenting cerebral metabolomic profiles during different anesthesia regimens; and study procedures are described in detail in Jacob et al. (2012). Briefly, after IRB approval [Committees on Research Involving Human Subjects (CORIHS), Stony Brook University] and parental consent, children (2–7 years) were anesthetized with sevoflurane (*N* = 37) or propofol (*N* = 50) and underwent routine MRI imaging for clinical evaluation. Common clinical indications for the diagnostic MRI scans included seizures, headache, and potential developmental delay (Jacob et al., 2012). Exclusion criteria were acute brain trauma, stroke or hemorrhage or any confirmed diagnosis of elevated intracranial pressure (Jacob et al., 2012). Scanning was performed on a 3.0T Philips Achieva whole body scanner, and high resolution T1-weighted and single voxel ^1^H MRS were performed in each session. A T1-weighted turbo field echo sequence was acquired in the sagittal plane at voxel dimensions of 0.94 × 0.94 × 1.00 mm.

### Data analysis

For gray matter and white matter analysis, the T1-weighted scans were segmented using SPM8 (Ashburner and Friston, 2000). The ^1^H MRS single-voxel point-resolved sequence (PRESS) was acquired in the cortex (e.g., parietal or temporal lobes) with following parameters voxel size of 1.5 × 1.5 × 1.5 cm^3^, TR/TE/2000 ms/32 ms, receiver bandwidth = 1200/2000 Hz, number of points = 1024/2048, and averages = 256. In the current analysis, the following metabolite concentrations were quantified and extracted from the spectra by linear combination of model (LCModel) analysis using the water concentration as an internal reference (Provencher, 2001): N-acetylaspartate (NAA) + N-acetylaspartylglutamate (NAAG) = tNAA; phosphorylcholine + glycerophosphorylcholine = total choline (tCh); creatine + phosphocreatine = total creatine (tCr); glutamate + glutamine = tGlx, and lactate (Lac). Partial volume effect in the water concentration was also considered in the concentration calculation (Lee et al., 2013).

### Calculation of the cerebral metabolic rate of lactate

To assess the degree of CMR_glucose_-CMR_O2_ uncoupling consistent with the measured lactate levels and to compare with previous reported CMR_glucose_ and CMR_O2_ in early childhood, we calculated the cerebral metabolic rate of lactate, CMR_lac_, as the difference between the unidirectional transport of lactate into the brain (V_in_) and the lactate efflux (V_out_) from the brain, Equation 1 (Boumezbeur et al., 2010), i.e., it is net lactate carbon flux across the blood-brain barrier. CMR_lac_ is defined as the cerebral metabolic rate of either net lactate production or consumption of plasma lactate by the brain. The two parameters V_in_ and V_out_ can be calculated based on Equation 2, 3, and CMR_Lac_, [Lac]_p_, and Lac]_B_ can be calculated with Equation 4 when different values for lactate concentrations or V_MAX_ are used:

(1a) $$\begin{array}{c} CMR_{Lac}=V_{in}-V_{out} \end{array}$$

(1b) $$\begin{array}{c} CMR_{Lac}=-2\;({CMR}_{glucose}-{CMR}_{O2}/6) \end{array}$$

(2) $$\begin{array}{c} V_{in}=\;V_{MAX}\frac{{\left[Lac\right]}_{p}}{K_{T}+\;{\left[Lac\right]}_{p}+{\left[Lac\right]}_{B}} \end{array}$$

(3) $$\begin{array}{c} V_{out}=\;V_{MAX}\frac{{\left[Lac\right]}_{B}}{K_{T}+\;{\left[Lac\right]}_{p}+{\left[Lac\right]}_{B}} \end{array}$$

(4) $$\begin{array}{c} \text{CM}{\text{R}}_{\text{Lac}}={\text{V}}_{\text{MAX}}\left({\left[\text{Lac}\right]}_{\text{p}}-{\left[\text{Lac}\right]}_{\text{B}}\right)/\left({\text{K}}_{\text{T}}+{\left[\text{Lac}\right]}_{\text{p}}+{\left[\text{Lac}\right]}_{\text{B}}\right) \end{array}$$

Where [Lac]_p_ and [Lac]_B_ are the concentrations of lactate in arterial plasma and brain, respectively. Since we did not measure [Lac]_p_ in the children we set it to be 0, 1, or 2 mM for the calculations, which is a reasonable range for the estimate since the clinical guides report normal plasma [Lac] in children in the range of 0.5–2 mM (Agrawal et al., 2004) and none of the children in our study was acutely sick or suffering from chronic infections (Jacob et al., 2012). Lactate transport kinetic parameters V_MAX_ and K_T_ determined previously in adult brain of 0.4 μmol/g/min and 5.1 mM, respectively were used for the calculations (Boumezbeur et al., 2010). We also examined the impact of a 3, 5, and 10-fold higher V_MAX_, based on studies of neonatal rats demonstrating that transport kinetics are elevated in young rat brain when compared to adult brain (Cremer et al., 1979).

### Calculation of the effect of different substrates on the measured OGI

**Figure 2:**
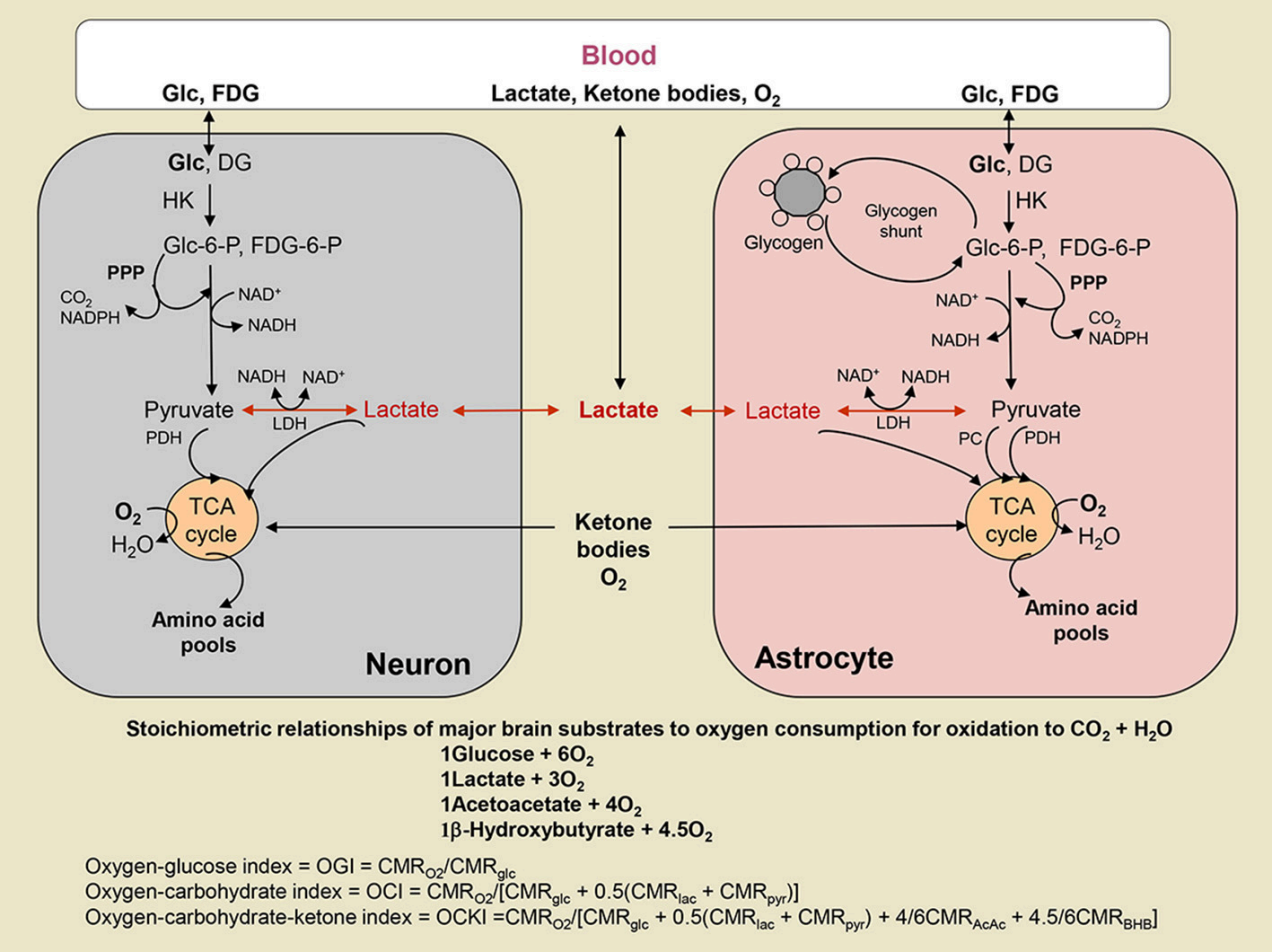
Oxygen-substrate stoichiometry in brain. Substrates are delivered to brain by blood where they are metabolized by brain cells that consume oxygen. Glucose (Glc) and the glucose analog fluorodeoxyglucose (FDG) are phosphorylated by hexokinase (HK) to their respective hexose-6-phosphate (P). FDG-6-P is trapped in the cell where phosphorylated, whereas Glc-6-P is further metabolized via the glycolytic pathway to a 3-carbon product that can be oxidized in mitochondria as either as pyruvate (Pyr) via pyruvate dehydrogenase (PDH) in all cells and pyruvate carboxylase (PC) in astrocytes, or as lactate (Lac) that may be taken up into mitochondria and converted to Pyr by mitochondrial lactate dehydrogenase (LDH) (see text and legend to Figure 1). Glc-6-P can also enter the pentose phosphate shunt pathway (PPP) to generate NADPH for management of oxidative stress or for use in biosynthetic reactions. The PPP also generates ribulose-5-P that is a precursor for nucleic acid synthesis and intermediates are rearranged to produce fructose-6-P and glyceraldehyde-3-P that can re-enter the glycolytic pathway and fructose-6-P may recycled into the PPP. In astrocytes, Glc-6-P is also stored as glycogen. Lactate can be released from brain when non-oxidative metabolism is upregulated more than the oxidative pathways. Lactate and ketone bodies can also be taken up from blood and oxidized, particularly in suckling mammals during development, as well as during exercise and starvation, respectively, when their blood levels rise. Different substrates consume different amounts of oxygen when completely oxidized, and the relationship between total oxygen consumption and total utilization of various substrates is illustrated. The oxygen-glucose index (OGI) is based on the stoichiometry of glucose oxidation, and assumes no other substrates are metabolized. The same OGI will be obtained whether Lac or Pyr is oxidized, as long as there is no uptake of these substrates from blood. Metabolism of other substrates is taken into account by the oxygen-carbohydrate (OCI) and oxygen-carbohydrate-ketone body (OCKI) indicies. Note that Lac and Pyr are converted to glucosyl units. Ketone bodies, acetoacetate (AcAc) and β-hydroxybutyrate (BHB), are metabolized in mitochondria.

Figure 2 shows the stoichiometries used in order to calculate the effect of different substrates on the measured OGI. We also give the equations (Figure 2; Table 4) for the oxygen-carbohydrate index (OCI) and oxygen carbohydrate ketone index (OCKI) which take into account the oxidation of lactate and lactate plus ketones, respectively, as opposed to only glucose in the OGI. As illustrated in Figure 2, oxygen consumed by complete oxidation of different substrates varies with the number of carbon atoms (and oxidation of β-hydroxybutyrate to acetoacetate before entering the TCA cycle), and it is necessary to take the stoichiometry into account when calculating molar oxygen/substrate ratios. When all substrates are included in the same calculation, the molar equivalent carbon each substrate is expressed relative to the oxygen consumed by glucose. For example, lactate and pyruvate would consume 3O_2_, and are equivalent to 0.5 glucose. Use of these calculations emphasizes the stoichiometry of net utilization of substrate(s) compared with oxygen, and has been used in OCI calculations during exercise to exhaustion (Quistorff et al., 2008; van Hall et al., 2009). Thus, if glucose is taken up into brain and converted to pyruvate that is transported into and oxidized in mitochondria or converted to lactate in cytoplasm, followed by its uptake and oxidation in mitochondria, the same number of moles of oxygen will be consumed per glucose. On the other hand, if lactate is released from brain or if non-oxidative metabolism predominates, the oxygen/substrate index falls below the theoretical maximum. In addition, if brain glycogen is consumed, as during hypoglycemia (Oz et al., 2009) or brain activation (Swanson et al., 1992; Cruz and Dienel, 2002), the additional carbon fuel must also be taken into account. In the present study, the children were anesthetized and glycogenolysis is not anticipated to be increased, since glycogen turnover in resting brain is very slow (Watanabe and Passonneau, 1973). However, glycogen may have contributed to brain metabolism during the CMR_glucose_ and CMR_O2_ assays, especially if the subjects were stimulated or stressed, and it would cause errors in calculated OGI.

### Statistical analysis

We analyzed associations between brain volumes and age using Analysis of Covariance (ANCOVA) with adjustment for gender. To examine the relation between LCModel-derived metabolite concentrations and age, an Analysis of Covariance (ANCOVA) with adjustment for anesthesia regimen (Sevoflurane or Propofol) and gender was employed. Analysis was conducted using XLSTAT (Version 2011.4.03).

## Results

### MRS spectral quality

**Figure 3:**
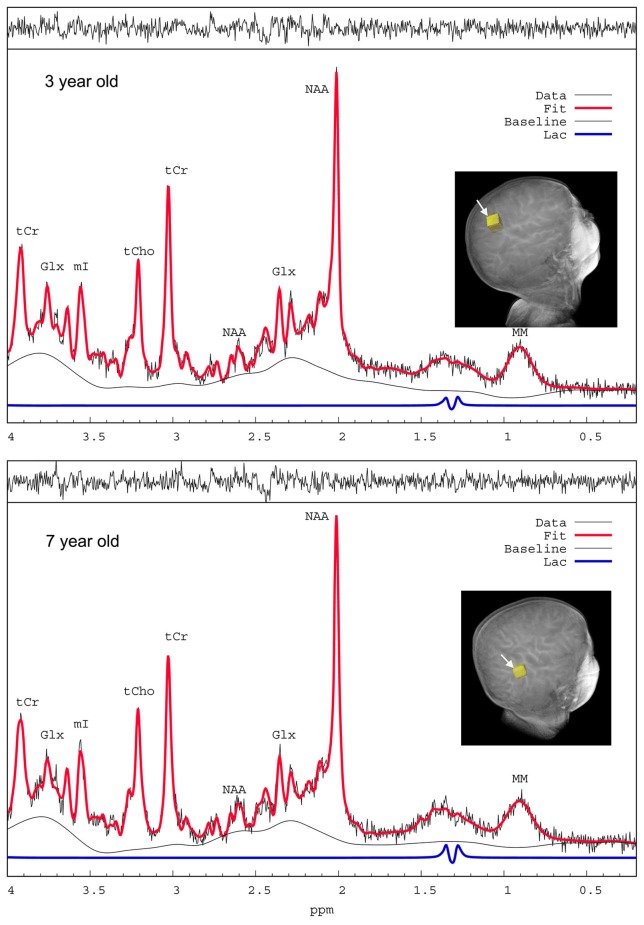
^1^H MRS spectra from a 3-year-old and a 7-year-old child. Representative proton magnetic resonance spectroscopic (^1^H MRS) spectra from parietal cortex of children anesthetized with sevoflurane and analyzed by LCModel. The spectra are of excellent quality with sufficient water suppression and spectral resolution to resolve at least 6–10 metabolites. The raw unsmoothed spectra are shown (black) in addition to the LCModel-fitted output (red solid lines). NAA, N-acetylaspartate; Glx, glutamate + glutamine; tCr, total creatine; mI, myo-inositol; tCho, total choline; MM, macromolecules. The LCModel- defined lactate peaks on the two spectra are shown in blue (scaled x4 for enhancing visualization of the peaks).

In order to assess spectral quality the ^1^H MRS spectra were checked for poor signal-to-noise ratio (SNR), spectral line width via full width at half maximum (FWHM) and baseline fluctuations estimated from LCModel analysis (Provencher, 2001), and 13 spectra were excluded. The average FWHM and SNR of the spectral NAA peaks were 0.028 ± 0.006 ppm and 22.3 ± 4.2, respectively indicating excellent spectral resolution and sensitivity. Figure 3 shows representative ^1^H MRS spectra from the cortex of a 3-year-old child (top) and a 7 year old child (bottom); and the LCModel-determined lactate peak is also depicted (blue, scaled × 4 for enhancing the peak). The Cramer–Rao lower bounds (CRLBs) which are the standard error estimates expressed in percent of the estimated concentrations (%SD) calculated by LCModel analysis (Provencher, 2001) for [tCr], [tNAA], [tCh] were 2–5%SD; and CRLB's for [tGlx] were 8–14%SD. The CRLB's for [Lac] were considerably higher and [Lac] <0.1 mM were discarded leaving 65 subjects with [Lac] for analysis, and the average CRLB for these was 80 ± 35%SD. The high CRLB for [Lac] was due to its low concentration in the brain being on the order of the noise level in some subjects.

### Brain morphometry and metabolites across early childhood (2–7 Years)

**Figure 4:**
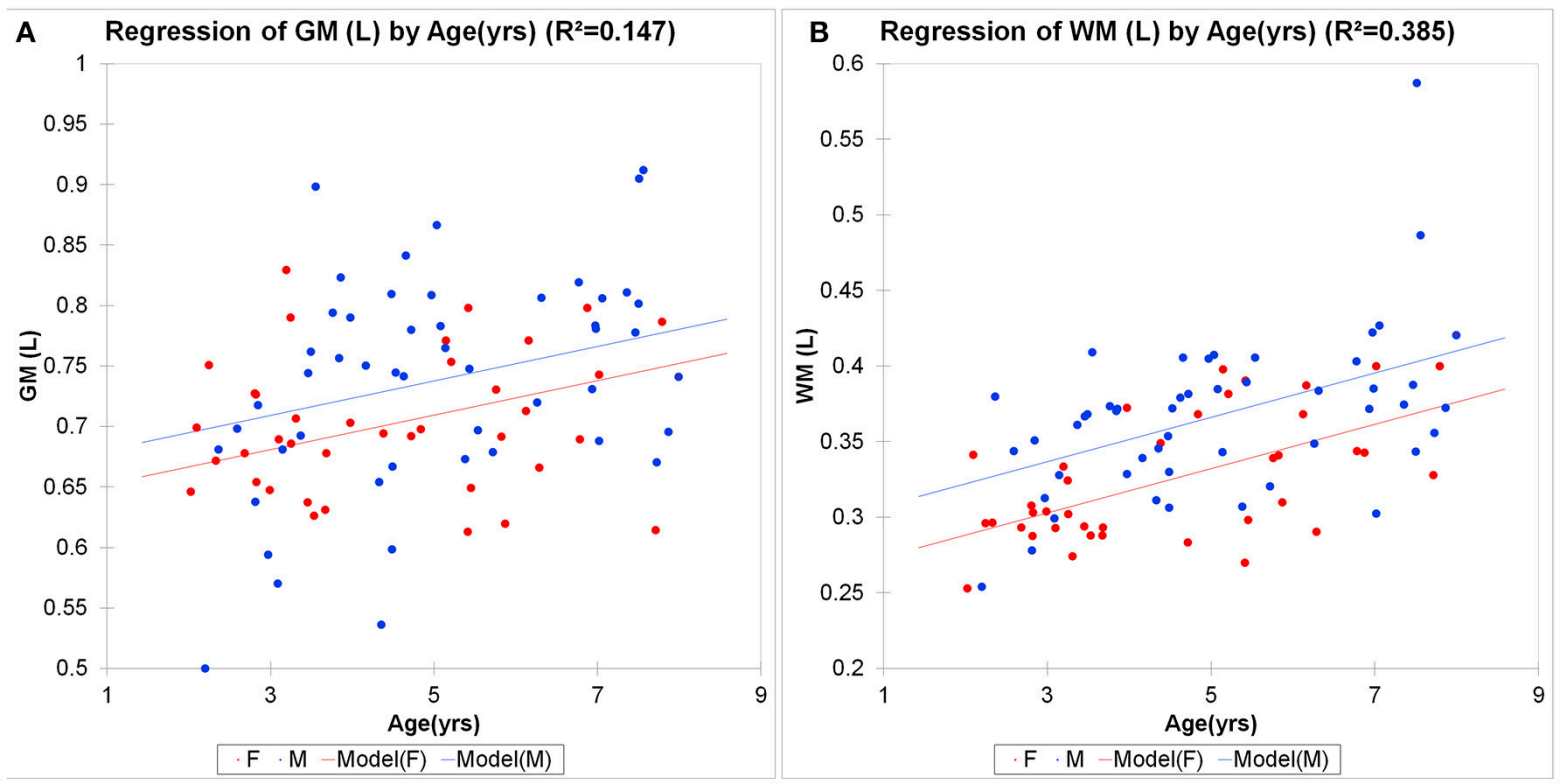
Morphometric brain analysis across childhood. Total brain gray **(A)** and white **(B)** matter volumes in male (M, blue circles) and female (F, red circles) children as a function of age. Linear regression analysis shows significant brain growth in gray matter (GM *R*^2^ = 0.147, *p* < 0.001; WM *R*^2^ = 0.385, *p* < 0.0001). For GM, 15% of the variability was explained by the two variables, with age being significant (*p* = 0.003) but not gender (*p* = 0.082). For WM, 39% of the variability was explained by the two variables, with age being more influential (*p* < 0.0001) compared to gender (*p* < 0.001).

Global brain morphometric analysis revealed that total gray matter (GM) and white matter (WM) in the children significantly correlated with age in the expected, positive direction (GM *R*^2^ = 0.14 WM *R*^2^ = 0.36, *p* < 0.001, Figure 4). For GM, 15% of the variability was explained by the two variables, with age being significant (*p* = 0.003) but not gender (*p* = 0.082). For WM, 39% of the variability was explained by the two variables, with age being more influential (*p* < 0.0001) compared to gender (*p* < 0.001). The concentration of tNAA ([tNAA], a neuronal marker) was in the range of 5–6 mM and also positively correlated with the children's age in agreement with a previous report (Kadota et al., 2001), but not with gender (Table 1). However, in contrast to [tNAA], none of the other metabolites including [tCr], [tCho], [Lac], or [tGlx] appeared to follow a linear age-dependency pattern (Table 1).

**Table 1:** Analysis of energy metabolites by age with gender and anesthesia regimen as covariates.

		**[Lac]**	**[tCho]**	**[tNAA]**	**[tCr]**	**[tGlx]**
*R*2		0.021	0.030	0.180	0.061	0.034
F		0.437	0.862	6.066	1.801	0.970
*P*-value		0.727	0.464	**0.001**	0.153	0.411
Age	F	0.513	0.474	7.459	0.007	2.133
	*P*-value	0.477	0.493	**0.008**	0.935	0.148
Gender	F	0.354	2.311	1.538	0.040	0.657
	*P*-value	0.554	0.132	0.218	0.841	0.420
Anesthesia	F	0.122	0.062	10.281	5.355	0.120
	*P*-value	0.728	0.803	**0.002**	**0.023**	0.730

*ANCOVA (Analysis of COVAriance) was used to analyze interactions between brain metabolites and age and anesthesia regimen. Given the R^2^ for [tNAA], 18% of the variability of the dependent variable—[tNAA]—is explained by the three explanatory variables. Among the explanatory variables, the anesthesia regimen and age are the most influential. Bold values are statistically significant*.

### Trajectory of brain lactate in early childhood

**Figure 5:**
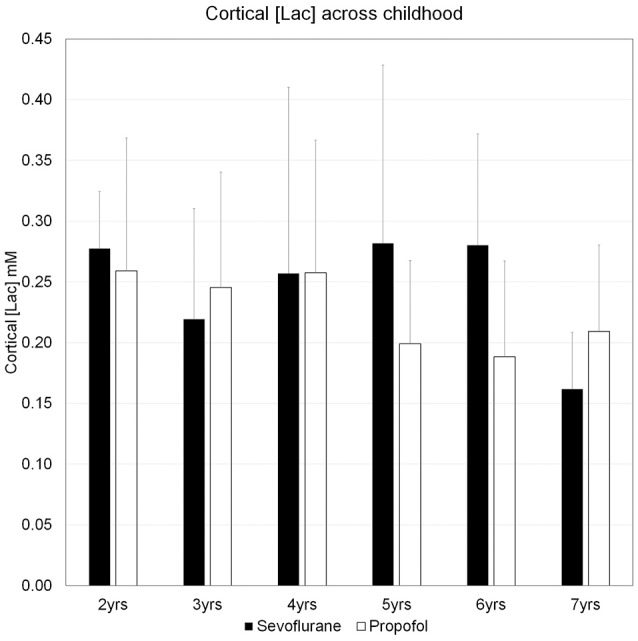
The concentrations of cerebral cortical lactate in children aged 2–7 years. Concentrations of lactate, [Lac], for each year of children aged 2–7 years, anesthetized with either sevoflurane or propofol are means + SD. For sevoflurane the ranges of [Lac] are given below: Age 2 years: 0.24–0.35 mM; Age 3 years: 0.13–0.37 mM; Age 4 years: 0.12–0.54 mM; Age 5 years: 0.15–0.51 mM; Age 6 years: 0.16–0.39 mM; Age 7 years: 0.11–0.20 mM. The number of subjects in each age group for the two anesthetics are as follows: Sevoflurane group: Age 2 (*N* = 5); Age 3 (*N* = 5); Age 4 (*N* = 6); Age 5 (*N* = 5); Age 6 (*N* = 6); Age 7 (*N* = 3). Propofol group: Age 2 (*N* = 5); Age 3 (*N* = 10); Age 4 (*N* = 6); Age 5 (*N* = 7); Age 6 (*N* = 4); Age 7 (*N* = 3). Please note that “Age 2,” children ≥2 yrs, <3 yrs; “Age 3 yrs,” children ≥3 yrs, <4 yrs; “Age 4 yrs,” children ≥4 yrs, <5 yrs; “Age 5 yrs,” children ≥5 yrs, <6 yrs; “Age 6 yrs,” children ≥6 yrs, <7 yrs; “Age 7 yrs,” children ≥7 yrs, <8 yrs.

**Figure 6:**
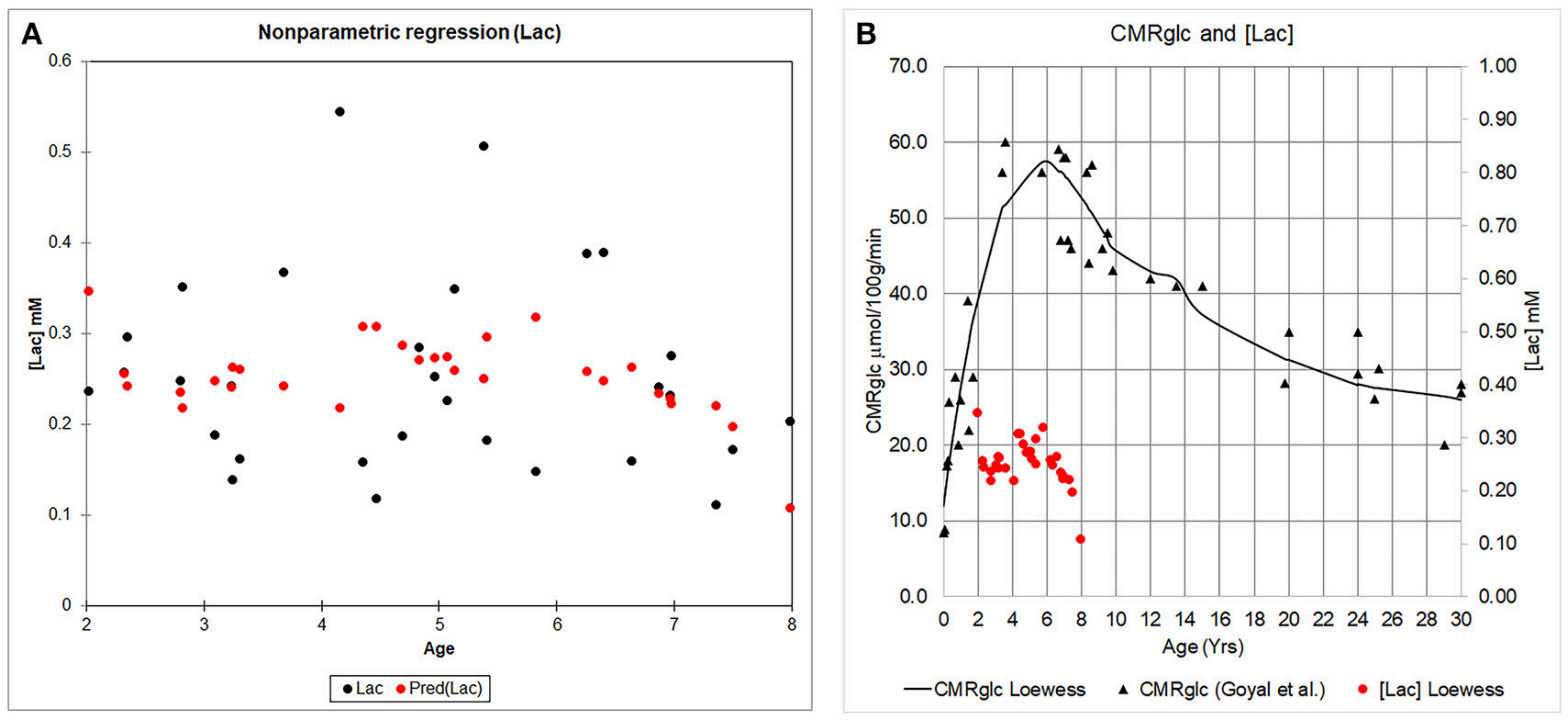
The concentration of cerebral cortical [Lac] and CMR_glucose_ across childhood. **(A)** Cerebral cortical [Lac] from children anesthetized with sevoflurane is plotted as a function of age (black circles). A Lowess regression (locally weighted regression and smoothing scatter plot) was fitted to the data using XLSTAT (Version 18.07); and is represented by the red circles. **(B)** Whole brain CMR_glucose_ data as reported by Goyal et al. (2014) (black triangles) is shown in relation to the Lowess fit of the [Lac] data (red circles).

We characterized the trajectory of the brain concentration of lactate, [Lac] across the children's ages, because previous reports documented enhanced levels of non-oxidative metabolism of glucose in early childhood and the peak excess CMR_glucose_ over CMR_O2_ occurred at 3–5 years of age (Goyal et al., 2014). Figure 5 shows the mean cortical [Lac] for each year of children aged 2–7 years, anesthetized with either sevoflurane or propofol and demonstrates that in all children, regardless of age and anesthetic, [Lac] is <1 mM. Further, we did not observe a [Lac]_B_ peak at ~ at 3–5 years, however, [Lac]_B_ in children anesthetized with sevoflurane was noted to be highest at ~5 years of age and reached a level of 0.28 ± 0.20 mM. Thus, mean cortical [Lac] in children is lower than the reported [Lac] values in brain of unanesthetized adults (0.5–1.0 mM) (Prichard et al., 1991; Bednarik et al., 2015; Rowland et al., 2016). Second, to explore the age-dependent relation with the AG trajectory (Goyal et al., 2014), we performed a Lowess, non-parametric regression of [Lac] from children anesthetized with sevoflurane which is shown in Figure 6A.

### Calculation of CMR_Lac_ and quantitative evaluation of its contribution to elevated non-oxidative metabolism of glucose

Using the standard reversible Michaelis-Menten model for brain lactate transport (Simpson et al., 2007; Boumezbeur et al., 2010) we calculated the magnitude and direction of brain lactate transport. Table 2 presents the calculated CMR_Lac_ for brain [Lac] for 4 year old children anesthetized with sevoflurane [average brain [Lac] was 0.28 ± 0.20 mM (range: 0.12–0.54 mM)]. Using previously measured plasma lactate concentrations in children of ~1 mM (Agrawal et al., 2004) and the kinetic constants for lactate transport measured previously in adults the calculated value of CMR_Lac_ was for net entry into the brain at a relatively low rate (0.04 μmol/g/min). The lactate that entered the brain would be oxidized, raising CMR_O2_ and causing errors in calculated OGI that does not account for lactate oxidation, as does the oxygen-carbohydrate index (OCI, Figure 2, Table 4). A net efflux of lactate only occurred if plasma lactate were assumed to be 0 mM and also would be at a very low rate (−0.02 μmol/g/min, Table 2) and much lower than the lactate efflux rate needed to account for the mismatch between glucose uptake and oxygen consumption derived by Goyal et al. of −0.36 μmol/g/min.

**Table 2:** Calculated values for CMR_Lac_, [Lac]_p_, and [Lac]_B_.

**Calculated value**	**Fixed values**				
	**CMR_Lac_ (μmol/g/min)**	**[Lac]_p_ (mM)**	**[Lac]_B_ (mM)**	**V_MAX_ (μmol/g/min)**	**K_T_ (mM)**
CMR_Lac_ = −0.02	–	0	0.28	0.4	5.1
CMR_Lac_ = +0.05	–	1	0.28	0.4	5.1
CMR_Lac_ = +0.09	–	2	0.28	0.4	5.1
CMR_Lac_ = −0.06	–	0	0.28	1.2	5.1
CMR_Lac_ = +0.14	–	1	0.28	1.2	5.1
CMR_Lac_ = +0.23	–	1	0.28	2.0	5.1
CMR_Lac_ = +0.45	–	1	0.28	4.0	5.1
[Lac]_B_ = 46	−0.36	0	–	0.4	5.1
[Lac]_B_ = 65	−0.36	1	–	0.4	5.1
[Lac]_B_ = 84	−0.36	2	–	0.4	5.1
[Lac]_B_ = 2.6	−0.36	1	–	2	5.1
[Lac]_B_ = 4.0	−0.36	2	–	2	5.1

*Values were calculated with Equation 4: CMR_Lac_ = V_MAX_ ([Lac]_p_ - [Lac]_B_)/(K_T_ + [Lac]_p_ + [Lac]_B_). The present study measured brain lactate concentration ([Lac]_B_) = 0.28 mM in 5-year-old sevoflurane anesthetized children (Figure 5), but their plasma lactate concentrations ([Lac]_p_), the kinetic constants for lactate transport across the blood-brain barrier (V_MAX_ and K_T_), and the rate of lactate utilization (CMR_Lac_) are not known. Two questions were, therefore, posed: (1) What is calculated CMR_Lac_ based on measured brain [Lac] and different values for plasma lactate concentration and V_MAX_? (2) What is brain lactate level when CMR_Lac_ is fixed and plasma lactate level and V_MAX_ are varied? Positive or negative values for CMR_Lac_ denote net influx or efflux of lactate into or from brain, respectively. Measured values for V_MAX_ (0.4 μmol/g/min) and K_T_ (5.1 mM) in normal adult human brain are from Boumezbeur et al. (2010) Because V_MAX_ is higher in developing rodent brain (but not known in human children), values were increased 3, 5, or 10-fold for the calculations. Note: (i) net lactate uptake occurs when plasma lactate level exceeds that in brain, and lactate efflux occurs when brain lactate level exceeds that in plasma, and (ii) calculated values for brain lactate levels based on adult kinetic constants are not realistic*.

To assess the impact of the kinetic constants used from adult brain on the calculations we also examined the impact of increasing the Vmax for lactate transport by a factor of 3, 5, and 10. At typical plasma lactate levels of 1 mM the calculated CMR_lac_ increased but the directionality (into the brain) remained the same. Based on studies in animal models (Cremer et al., 1979) the maximum anticipated increase in lactate transport in children was 3-fold and assuming 0 plasma lactate the efflux of lactate would only be −0.07 μmol/gm/min which again is well-below the predicted −0.36 μmol/g/min. Note that, based on Equation 4 lactate efflux can only occur when [Lac]_B_ exceeds [Lac]_p_.

In order to assess the concentration of brain lactate which would be required to account for the reported mismatch we calculated brain lactate concentration [Lac]_B_ for a CMR_lac_ of −0.36 μmol/g/min (Table 2). Using the V_MAX_ measured in adults and varying the plasma lactate concentration from 0 to 2 mM yielded a predicted brain [Lac] ranging from 46 to 84 mM. Even with a 5-fold increase in V_MAX_ assumed the brain [Lac] would have to be between 2.6 and 4.0 mM which is 8–12 times higher than the measured value.

### Re-calculating CMR_glucose_ and OGI across childhood using updated values for the lumped constant

Because brain lactate levels and calculated lactate efflux rates based on our data were too low to explain the low OGI reported in children (Goyal et al., 2014), we considered and evaluated an alternative explanation for low OGI. The FDG-PET literature has reported and discussed updated values for the lumped constant (LC), the factor that accounts for kinetic differences in rates of transport and phosphorylation between FDG and glucose and is used to convert [^18^F]FDG phosphorylation rate to CMR_glucose_. Based on our review of the Supplemental Table 1 of Goyal et al. (2014) the CMR_glucose_ data were taken from Table 1 of Chugani et al. (1987) in which a LC of 0.42 was used for subjects of all ages as originally published for adult brain by Phelps and coworkers (Phelps et al., 1979; Huang et al., 1980). Since that time higher values for adult brain have been found with recent values close to 0.8–0.85 (Graham et al., 2002; Hyder et al., 2016).

Due to the uncertainty regarding the true value of the LC, we re-calculated the CMR_glucose_ using LC = 0.65, a value subsequently determined in the Phelps laboratory for adult brain (Wu et al., 2003) that also determined LC = 0.42, the value used by Chugani et al. (1987), as well as LC = 0.80, a value determined by Hyder et al. (2016) that is within the range of the higher values noted above. We performed the calculations based on the peak CMR_glucose_ = 0.58 μmol/g/min and OGI = 4.1 in the loessR plot in Figure 2A of Goyal et al. (2014). When LC = 0.80 was used, CMR_glucose_ fell and approached CMR_glucose_ for normal adults (Table 3). Importantly, the OGI increased from 4.1 to 6.4 and 7.9 when higher values for the LC were used, and the magnitude of non-oxidative metabolism of glucose representing ~33% CMR_glucose_ in excess of CMR_O2_ was reversed. When LC was increased by 55% from 0.42 to 0.65, CMR_O2_ and CMR_glucose_ were nearly stoichiometrically matched because the calculated CMR_glucose_ was reduced by a corresponding percentage (Table 3). There was no excess glucose consumed and the predicted lactate uptake agrees with calculated CMR_Lac_ of +0.04 μmol/g/min based on measured brain [Lac] (Figures 5, 6; Table 2).

**Table 3:** Estimates of changes in OGI and lactate efflux rates from brain of children when updated values for the lumped constant are used to calculate CMR_glucose_.

**Lumped constant**	**CMR_glucose_ (μmol/g/min)**	**OGI**	**CMR_O2_/6 (μmol/g/min)**	**CMR**_**O2**_**-CMR**_glucose_ **stoichiometric mismatch**	
				**CMR_glucose_-(CMR_O2_/6) (μmol/g/min)**	**Equivalent CMR_Lac_ (μmol/g/min)**
0.42	0.58	4.1	0.4	0.18	−0.36
0.65	0.37	6.4	0.4	−0.03	+0.06
0.80	0.30	7.9	0.4	−0.10	+0.20

*Analysis of the stoichiometric mismatch between oxygen and glucose was evaluated using equation 1b: CMR_Lac_ = −2[CMR_glucose_ - (CMR_O2_/6)] for different values for the lumped constant that alter calculated CMR_glucose_. CMR_Lac_ is assigned a negative value to denote release of glucose equivalents from brain when CMR_glucose_ exceeds CMR_O2_; positive values are obtained when CMR_O2_ exceeds CMR_glucose_ and indicate oxidation of other substrates (carbohydrate or ketone bodies) that were not taken into account in the OGI calculation. Maximal values of CMR_O2_ and CMR_glucose_ in the loessR plots for ~3–5 year old children in Figure 2A of Goyal et al. (2014) i.e., 2.4 and 0.58 μmol/g/min, respectively, were used to calculate OGI = CMR_O2_/ CMR_glucose_. CMR_glucose_ for updated values of the lumped constant was calculated by multiplying CMR_glucose_ = 0.58 by 0.42/0.65 or 0.42/0.80 and used to calculate updated OGIs. Stoichiometric balance between CMR_glucose_ and CMR_O2_ was determined by subtracting the glucose equivalents of maximal CMR_O2_ (i.e., CMR_O2_/6 = 2.4/6 = 0.4) from CMR_glucose_. For comparison to values in brain of normal resting awake adults, OGI determined in 8 independent studies by arteriovenous differences, was 5.95 ± 0.27 (mean ± SD) (Quistorff et al., 2008). This value for OGI was determined by a method independent of the value of the lumped constant. Whole-brain CMR_glucose_ in normal resting awake adult human brain was 0.26 ± 0.07 μmol/g/min when calculated using the lumped constant = 0.80, whole brain CMR_O2_ was 1.36 ± 0.37 μmol/g/min, and whole-brain OGI was 5.17 + 0.95 (Hyder et al., 2016)*.

Goyal et al. (2014) strongly emphasized the temporal profile of enhanced non-oxidative metabolism of glucose (higher CMR_glucose_ compared with CMR_O2_) in children 1–10 years of age, with a peak at about 5 years of age (as illustrated in Figure 6B). However, due to the uncertainty in the true value for the LC and its high impact on OGI and therefore on the magnitude of non-oxidative metabolism of glucose revealed by calculations as illustrated in Table 3, we recalculated the CMR_glucose_ trajectories with updated values for the LC along with CMR_O2_. Figure 6A shows the age-dependent changes for the Goyal data for CMR_glucose_ (blue) and CMR_O2_ (red, expressed in glucose equivalents as calculated by Goyal et al., CMR_O2_/6, a calculation that assumes all oxygen consumed is due to glucose oxidation), and for re-calculated values with LC = 0.65 (green), and LC = 0.80 (brown). When higher LC values were used the discrepancy between CMR_glucose_ and CMR_O2_ was age-dependent, with CMR_O2_ exceeding CMR_glucose_ in 1–2 year old children, and nearly-stoichiometric rates at ages 5–10 years (Figure 6A).

## Discussion

In this study we measured brain [Lac] in 65 children across 2–7 years and documented that [Lac]_B_ on average was <0.3 mM throughout and below previous MRS measurements in the adult brain (0.5–0.7 mM). In addition, [Lac]_B_ did not peak at 3–5 years inconsistent with the peak excess CMR_glucose_ over CMR_O2_ and low OGI documented at 3–5 years of age (Goyal et al., 2014), which they ascribed to the needs of increased synaptogenesis. However, there are other potential reasons for the fall in OGI, including lactate release from brain. This possibility was ruled out because the brain [Lac] we measured was many fold below what is needed to explain the quantitative drop in the OGI and was consistent with small net brain uptake as opposed to efflux of lactate. We discuss these findings below in light of what is known about fuel consumption in the developing brain and evaluate potential metabolic and methodological explanations for the discrepancy between the reported low OGI and the brain [Lac] measured. Previous studies have discussed the quantitative contribution of lactate uptake into resting adult brain (Boumezbeur et al., 2010), the oxygen/substrate stoichiometry in brain of non-stimulated, sedentary human subjects (Hyder et al., 2016), decreases in the ratio during brain activation (Dienel and Cruz, 2016), and the contributions of glucose and lactate during exhaustive exercise (Quistorff et al., 2008; van Hall et al., 2009). The present study examines the basis for decreases in this ratio in brains of children during development.

### Enhanced aerobic non-oxidative metabolism of glucose in the developing brain and relation to brain lactate

To assess whether lactate efflux could account for the low OGI reported in early childhood we calculated CMR_Lac_ based upon the measured concentration of brain lactate and literature values for plasma lactate concentration and transport kinetics. As shown in Table 2 these calculations indicate that based on the measured brain [Lac] an inflow of plasma lactate is predicted. In order to obtain lactate efflux sufficient to account for the reported elevated non-oxidative metabolism of glucose (and low OGI) brain [Lac] ranging from 46 to 84 mM (with the range based upon the concentration of plasma lactate and adult brain kinetic constants) were calculated which is two orders of magnitude above the measured values.

An alternate possibility to explain our data in relation to previously-reported data (Goyal et al., 2014) is that children have several-fold higher lactate transport activity through the monocarboxylate transporter (MCT) system than adults. Preclinical data in rodents show that the expression of MCTs is higher in neonates than in adults (Gerhart et al., 1997). Cremer et al. measured MCT transport in neonatal and adult rats and the transport kinetics were found to be ~3-fold higher in the neonates (Cremer et al., 1979). Assuming that V_MAX_ is 5-fold higher we calculated a minimum brain [Lac] needed to account for elevated non-oxidative metabolism of glucose of 2.6 mM which, is 9-fold greater than the measured values. Using the measured value of brain [Lac] the impact of a higher V_MAX_ would be to increase lactate influx (Table 2). We note that a 3-fold higher value is most likely, well-above the elevation, if any, in the children studied since it was obtained from rat pups that were not yet weaned, during which time there is a much higher percentage of ketones and other monocarboxylic acid substrates consumed by the brain (Chowdhury et al., 2007).

Overall our ^1^H MRS data - which were not supportive of lactate efflux from children's brain - are in agreement with previous data reporting a cerebral arterio-venous (AV) difference for lactate of ~0 in seven anesthetized children (Persson et al., 1972). In another study which documented AV-differences of glucose and oxygen in children, OGI was close to the expected theoretical value of 6:1 (Settergren et al., 1976) (see ketones as alternate fuels and Table 4, below).

**Table 4:** Blood flow, metabolic rates, and calculated oxygen/substrate utilization ratios and brain lactate concentration in brain of young children.

**Reference**	**1^a^**	**2^b^**		**3**^d^	**4^e^**	**5^f^**	**6^g^**	**7^h^**	**8^i^**				
Age range (years)	0.5–3.3	3–11	Adults	0.58–14	0.08–0.58	10–15	1–15	21–24 55–65	0–1	1–2	3–8	9–15	19–30
Mean age (years)	0.92	6.1	24.5			12							
Number of subjects	10	9	12	7	17	7	42–65	10	7	4	12	6	7
Duration of fasting (h)	3				9		10	15	4	4	4	4	4
Physiological status during assay	Awake, 15% N_2_O	Awake, 15% N_2_O	Awake, 15% N_2_O	70%N2O	awake	75 → 50% N_2_O	75 → 50% N_2_O	awake	All groups awake				
Anesthesia duration (min)							20–40						
CBF (ml/g/min)	0.903	1.064	0.601				0.68	0.64					
Arterial conc. (μmol/ml)													
Glucose Lactate Pyruvate Acetoacetate β-hydroxybutyrate	1.80 0.094			4.18 1.52 0.08 0.60 1.75	5.21 1.25 0.12 0.28		5.27 1.13 0.104 0.273 0.890	4.57 0.61 0.065 0.159 0.347					
(A-V) (μmol/ml)													
O_2_ Glucose Lactate Pyruvate Acetoacetate β-hydroxybutyrate	2.87 0.726 −0.507 −0.0085			1.49 0.29 −0.03 −0.01 0.08 0.09	2.23 0.48 −0.27 0.03 0.04	2.08 0.33 0.012 0.014	2.17 0.36 −0.07 −0.01 0.018 0.049						
CMR (μmol/g/min)													
O_2_ Glucose Lactate Pyruvate Acetoacetate β-Hydroxybutyrate	2.59 0.656 −0.458 −0.008	2.31^c^	1.86				1.35 0.248 −0.048 −0.008 0.012 0.034	1.72 0.248 −0.028 −0.004 0.004 0.006	0.204	0.274	0.481	0.392	0.243
Respiratory quotient	1.00	0.97	0.94										
OGI	3.95			5.14	4.65	6.3	6.03	6.94	11.3^j^ 6.62^k^	8.4^j^ 4.93^k^	4.8^j^ 2.81^k^	5.9^j^ 3.44^k^	7.7^j^ 6.91^k^
OCI	6.13			5.52	6.46	6.3	6.78	7.41					
OCKI				3.81	4.56	4.04	5.88	7.19					
Calculated O_2_ uptake	2.81			2.35	2.37	2.09	2.21	1.44					
%O_2_ from ketone oxidation				30.9	12.7	5.3	13.2	3.0					
Lac+Pyr release (% Glc)	−35.5			−6.9	−28.1	0	−11.3	−6.5					
Calc.[Lac]_B_: V_MAX_ = 0.4 V_MAX_ = 2.0 V_MAX_ = 4.0	−64.8 4.4 2.9						2.14 1.31 1.22	1.08 0.70 0.65					

Values are means; those not included were not determined/reported by the tabulated studies.

CBF, cerebral blood flow; (A-V), arteriovenous difference that is positive when there is net uptake into brain and negative when there is net efflux; Glc, glucose; Lac, lactate; Pyr, pyruvate; AcAc, acetoacetate; BHB, β-hydroxybutyrate; KB, ketone bodies (AcAc + BHB); CMR, cerebral metabolic rate; OGI, oxygen-glucose index; OCI, oxygen-carbohydrate index; OCKI, oxygen-carbohydrate-ketone index.

**Metabolic rates and oxygen/substrate ratios:**

CMR_substrate_ = CBF(A-V)_substrate_.

OGI = CMR_O2_/CMR_glucose_ = (A-V)_O2_/(A-V) _glucose_ and assumes no other substrates are oxidized.

OCI = CMR_O2_/[CMR_glucose_ + 0.5(CMR_lac_ + CMR_pyr_)] = (A-V)_O2_/[(A-V) _glucose_ + 0.5((A-V)_lac_ + (A-V)_pyr_)], where pyruvate and lactate are expressed in glucose equivalents 1 glucose = 2 pyruvate or 2 lactate. OCI takes into account lactate + pyruvate uptake or efflux from brain and assumes no other substrates are consumed, which is generally valid for normal, non-fasted adults during rest or graded exercise to exhaustion.

OCKI = CMR_O2_/[CMR_glucose_ + 0.5(CMR_lac_ + CMR_pyr_) + 4/6CMR_AcAc_ + 4.5/6CMR_BHB_] = (A-V)_O2_/[(A-V) _glucose_ + 0.5((A-V)_lac_ + (A-V)_pyr_) + 4/6(A-V)_AcAc_ + 4.5/6(A-V)_BHB_], where utilization of other substrates are expressed in glucose equivalents for oxygen utilization. Oxidative metabolism of one glucose, one AcAc, or one BHB molecule consumes 6, 4, or 4.5 molecules of O_2_ (Hawkins et al., 1971). The OCKI calculation accounts for the major substrates consumed or released from brain relative to oxygen.

**Ketone body (KB) oxidation as percent of calculated O**_2_**utilization:** Calculated O_2_ uptake = 6*[(A-V)_glc_+0.5((A-V)lac + (A-V)_pyr_)] + 4(A-V)_AcAc_ + 4.5(A-V)_BHB_, assuming complete oxidation of ketone bodies. Calculated %KB oxidation = 100*(4(A-V)_AcAc_+4.5(A-V)_BHB_)/calculated (A-V)_O2_. For adults, CMR values replaced (A-V) in the equations.

**Lactate-pyruvate release/glucose uptake:** Lactate + pyruvate release/uptake from brain (negative value if release) is expressed in glucose equivalents as % of glucose uptake = 100*0.5[(A-V)_lac_ + (A-V)_pyr_]/(A-V)_glc_.

**Respiratory quotient (RQ):** RQ = volume of CO_2_ produced/volume of O_2_ consumed = (A-V)_CO2_/(A-V)_O2_. An RQ of 1.0, 0.8 or 0.7 indicates that carbohydrates, proteins, or lipids/ketones, respectively, are metabolized, with intermediate values indicating mixed fuel utilization.

**Calculated brain lactate concentrations:** [Lac]_B_ was calculated (Calc.) with Equation 4: CMR_Lac_ = V_MAX_ ([Lac]_p_ - [Lac]_B_) /(K_T_ + [Lac]_p_ + [Lac]_B_) using measured [Lac]_p_ and CMR_lac_ and different values for V_MAX_. Positive or negative values for CMR_Lac_ denote net influx into or net efflux of lactate from brain, respectively. Measured values for V_MAX_ (0.4 μmol/g/min) and K_T_ (5.1 mM) for plasma-brain lactate transport in normal adult human brain are from (Boumezbeur et al., 2010). For calculations V_MAX_ values 5 or 10 times higher were also used because based on rodent studies the developing brain may have a higher V_MAX_ for blood-brain barrier lactate transport (Cremer et al., 1979).

a Most children cried and required some restraint during the procedure, especially at time of needle punctures. PCO_2_ did not change significantly during the procedures.

b The authors stated that great pains were taken to minimize anxiety in the children, including having the dim lighting, minimal stimulation, and providing a movie on the ceiling that was considered to be unlikely to influence the global CBF or CMRO_2_. Low anxiety is supported by recording of mean pulse rates and mean arterial blood pressures were in the range of normal, resting 6-year-old children. Also, one child that had 4 repeated determinations with no significant changes due to familiarity with the procedure. The authors' subjective opinion was that the children were less anxious than the adults.

c Global CMR_O2_ had no significant correlation, positive or negative, with age between age 3 to 10 years.

d Children were pre-medicated with morphine and atropine, anesthesia induced with thiopentane, intubated, ventilated with 70% N_2_O/30%O_2_. Data for mild hypercapnia were also reported but not tabulated.

e Data for infants <0.15 years old were also reported but not tabulated. If the infants completely oxidized the ketone bodies they would account for 13% of total oxygen consumption, with glucose corrected for lactate efflux accounting for 87%.

f Children were pre-medicated with morphine, anesthesia induced with thiopentane, general anesthesia with pancuronium (prior to intubation) and 75% N_2_O/25%O_2_ that was abruptly reduced to 50% N_2_O during the CBF assay. More detailed data for infants <1 year old were also reported but not tabulated. In these infants, blood ketone body concentrations were much higher than in 12-year-old children, and (A-V) differences for AcAc and BHB were greater in the infants in whom net ketone body uptake accounted for 13% of measured oxygen uptake, assuming complete oxidation. However, less oxygen was consumed compared to calculated oxidation of glucose corrected for lactate + pyruvate release and ketone bodies, and this discordance could not be explained. Infants released lactate + pyruvate from brain to blood, equivalent to 6% of glucose uptake. Equally-detailed assays were not reported for the children.

g Children were pre-medicated with morphine and atropine, anesthesia induced with thiopentane, general anesthesia with pancuronium and 75% N_2_O/25%O_2_ that was abruptly reduced to 50% N_2_O during the CBF assay. (A-V) Differences were measured in 42 children (age range 1–15 years old), whereas CBF and O_2_ were measured in more children, including those at younger ages. Uptake of the ketone bodies was positively correlated with arterial concentration. CBF and the metabolic rates for oxygen, glucose, lactate, pyruvate, acetoacetate, and β-hydroxybutyrate were not correlated with age. However, the relationship between measured oxygen consumption and the amount needed for complete oxidation of glucose, acetoacetate, and β-hydroxybutyrate minus release of lactate + pyruvate was poor, as reported in Settergren et al. (1976); the basis for this finding is unknown. The authors reported considerable variability in CBF, so OGI, OCI, OCKI, and %lactate released were calculated from (A-V) differences. To calculate [Lac]_B_ for different V_MAX_ values it was necessary to use CMR_Lac_.

h Subjects were calm and relaxed after catheter insertion. Data were obtained for two groups of adults (21–24 and 55–65 years old; n = 5/group) that were not significantly different, and results were pooled.

i CMR_glucose_ is for cerebral hemispheres in children who had transient neurological events that did not significantly affect neurodevelopment and were considered to be reasonably representative of normal children. Some children had medication on the day of the study. Children that became drowsy during the assay were tapped on the shoulder but other stimuli were minimized. Regional values for CMR_glucose_ were also reported but are not tabulated.

j Global CMR_O2_ = 2.31 from Kennedy and Sokoloff (1957) was used to calculate OGI in awake children because no correlation with age (3–11 years old) was reported, whereas OGI in awake adults was based on CMR_O2_ = 1.86 determined in adults.

k Global CMR_O2_ = 1.35 from Settergren et al. was used to calculate OGI because no correlation with age was reported in anesthetized children (1–15 years old), whereas CMR_O2_ = 1.68 for awake adults was used to calculate OGI for adults. Settergren et al. also reported no age-related correlation of CMR_glucose_, CMR_Lac_, CMR_pyr_, CMR_AcAc_, or CMR_BHB_ across age between 1–15 years old in anesthetized children, contrasting the results of Chugani et al. (1987) in awake subjects. Note that global CMRO_2_ in 1–15-year-old anesthetized children the study by Settergren et al. (1980) is lower than that of awake adults the Lying-Tunell et al. (1980) and Kennedy and Sokoloff (1957) studies, whereas awake children age 3–11 years old had higher global CMRO_2_ than awake adults.

1. Mehta et al. (1977); 2. Kennedy and Sokoloff (1957); 3. Settergren et al. (1973); 4. Kraus et al. (1974); 5. Settergren et al. (1976); 6. Settergren et al. (1980); 7. Lying-Tunell et al. (1980); 8. Chugani et al. (1987).

### Alternate metabolic pathways to explain high non-oxidative metabolism of glucose in early childhood, a complex phenomenon

The concept of enhanced “aerobic glycolysis” (Goyal et al., 2014) (i.e., enhanced non-oxidative metabolism of glucose) is derived from consumption of more glucose than oxygen in the presence of abundant oxygen. The inference is that glycolytic flux is increased but the downstream fate of the glucose carbon is not established. Flux of glucose into many pathways could contribute to the CMR_O2_-CMR_glucose_ mismatch (Figure 1). We assess below possible contributions from these pathways.

#### Pentose phosphate pathway

One alternate possibility to explain the elevated non-oxidative metabolism of glucose is the pentose phosphate pathway. The use of glucose for biosynthesis involves both energy production, production of NADPH via the pentose phosphate pathway, and use of different pathways to incorporate glucose carbon into macromolecules that might be used for synaptic remodeling (Figure 1). Studies of the pentose phosphate pathway in adults (Baquer et al., 1988) suggest that it works primarily in the direction of NADPH production in which 1 carbon is lost per glucose that goes through the pathway with the remainder of the carbons reentering glycolysis and being converted to pyruvate and lactate. Therefore, even if all the glucose phosphorylated into glucose-6-phosphate (Glc-6-P) enters the pentose shunt it would only reduce the rate of glycolysis by 1/6 unless there is a very large ribose synthesis flux.

However, pentose phosphate pathway activity is higher during brain development (Baquer et al., 1977, 1988), and a greater fraction of glucose carbons may not enter glycolysis immediately [either being removed as riboses or lost through extensive cycling at the level of fructose-6-phosphate (Fru-6-P) which can be in relatively fast exchange with Glc-6-P via phosphoglucose isomerase (Rodriguez-Rodriguez et al., 2013); Figure 1] resulting in a larger underestimate of the CMR_O2_-CMR_glucose_ mismatch based on lactate production and levels. In fact, if recycling is complete, the shunt could explain most or all of the fall in OGI. The stoichiometry of the pentose shunt is 3 Glc-6-P → 3 CO_2_ + 2 Fru-6-P + 1 glyceraldehyde-3-phosphate (GAP). If *all of the Fru-6-P* is recycled by conversion to Glc-6-P that re-enters the shunt pathway, then one “new” Glc-6-P from glucose (or glycogen) is required per cycle, with the net result that for each glucose that enters as Glc-6-P, 3 CO_2_ + 1 GAP are produced. If the GAP is oxidized, the OGI would be 3 because half of the equivalents of the incoming glucose are converted to CO_2_ without oxygen consumption. If the GAP is converted to lactate and released from brain, OGI = 0. High activity of the pentose shunt in young children coupled with complete Fru-6-P recycling could explain both the low OGI and inability to account for the additional glucose carbon consumed in excess of oxygen because it is released as CO_2_. A caveat is that CO_2_ production without oxygen consumption via the pentose shunt would *increase* the respiratory quotient (RQ—see legend to Table 4 for definitions and discussion below) above 1.0, the value determined in young children that is indicative of carbohydrate utilization (Table 4). However, oxidation of ketone bodies has an RQ of 0.7, and the combination of high pentose shunt activity plus oxidation of blood-borne ketone bodies in brain of young children may explain the net RQ = 1. Future studies in children using ^13^C MRS technology to directly measure the pentose phosphate pathway and ketone body utilization could potentially distinguish these possibilities (Rothman et al., 2011).

#### Use of glucose carbons as biosynthesis precursors

In addition to ribose formation from the pentose phosphate pathway there are many other pathways by which carbons derived from glucose can be used for net biosynthesis, such as for lipids and amino acids. In order to assess this possibility, we calculated the approximate rate of increase in biomass implied by the non-oxidative metabolism of glucose mismatch using the following expression based on the “aerobic glycolysis” data of Goyal et al. (2014) (see Table 3)

Rate of biomass increase = the rate of “aerobic glycolysis” (μmol glucose/min/g brain)^*^brain weight

For the reported excess of glucose consumption over oxidation at 5 years old of 0.18 μmol/g/min and an average brain weight of 1300 g this calculation yields ~1800 g per month of additional carbon incorporation. This amount is well-over the total brain weight (which is ~70% water) and clearly not possible.

An alternate possibility, discussed by Goyal et al. (2014), is that there is a high level of synaptic turnover so that the carbon incorporated from glucose into nucleotides, lipids and proteins in the building of new synapses is largely matched by synapse breakdown and catabolism of the structural components. However, if this were the case then the released carbon building blocks would be oxidized at the same rate as new carbons are incorporated resulting in a normal OGI value.

#### Ketones and lactate as alternate fuels in early childhood

A limitation of the OGI is that it only takes into account the relationship between oxygen and glucose. The oxidation of non-glucose substrates is assumed to be negligible in adult brain, which may not be the case in children. Accurate values for the oxygen-fuel index would require measurement of net uptake into brain of all alternate substrates (e.g., β-hydroxybutyrate, acetoacetate, lactate) in plasma plus utilization of brain glycogen. It is well-known that in the developing brain lactate and ketones serves as fuel and substrates during the suckling period and beyond and ketones are essential for brain lipid synthesis (Settergren et al., 1976). For example, children 2–6 years of age have been reported to have significantly higher overnight fasting values of β-hydroxybutyrate and acetoacetate than older children and adults (Persson et al., 1972). Also, in a study where children were anesthetized (with N_2_O) the cerebral uptake of ketones (acetoacetate and β-hydroxybutyrate) accounted for ~13% of the measured oxygen uptake, assuming complete oxidation of the ketones (Settergren et al., 1976). In addition to ketones our calculations suggest that plasma lactate could be a net oxidative energy source in the developing brain, albeit at a low level. The effect of net lactate oxidation would be to increase the measured OGI due to the increase in oxygen consumption for the same amount of glucose uptake. As shown in Figure 2 and Table 4 alternate carbohydrate indicies can be defined that takes glucose, lactate, and ketone body net uptake into account (excluding brain glycogen consumption that cannot be measured in young children and is very difficult to measure in adults).

Table 4 summarizes results from metabolic studies in awake and N_2_O-anesthetized children and reveals the impact of inclusion of inclusion of lactate and ketone body fluxes on calculated oxygen/substrate ratios. If lactate efflux is not taken into account in study 1, OGI is too low compared with OCI (oxygen-carbohydrate index; see legend to Table 4 for definitions and equations for calculation). When lactate and ketone bodies are included in the calculation of OCKI (oxygen-carbohydrate-ketone index), the general trend is for OCI to exceed OGI due to correction of glucose uptake for lactate efflux in all studies where measured, then OCKI is falls below OCI due to inclusion of ketone body uptake (Table 4). In most cases, the calculated oxygen uptake is similar to the measured oxygen uptake value, and oxidation of ketone bodies accounted for 3–13% of total oxygen consumption except for study 3 where calculated oxygen uptake was 59% higher than the measured value and ketones accounted for 30% of oxygen uptake. Lactate *efflux* accounted for 0–11% of glucose uptake in the 0.6–15-year-old N_2_O-anesthetized children and 28–36% in children <3.3 years old when awake, suggesting stress-induced glycolysis/glycogenolysis and enhanced release. Of interest, OCI in study 1 is 6.1 and the respiratory quotient (RQ = (A-V)_CO2_/(A-V)_O2_) is 1.00 indicating strictly carbohydrate oxidation, whereas the RQ in older children was slightly <1, suggesting some ketone body use. (See discussion above regarding the potential balancing of pentose shunt and ketone body oxidation to influence the RQ).

#### Glycogen and the glycogen shunt

Little is known about the role of glycogen in energy metabolism during brain development (Rust, 1994). However, glycogen turnover with lactate release from brain in conjunction with synaptic activity would consume glucose without oxygen. Previous studies have considered the role of the glycogen shunt (i.e., the cycling of glucose-6-phosphate from the glycolytic pathway into glycogen and its return upon glycogenolysis) to explain, in part, the CMR_glucose_-CMR_O2_ mismatch observed during brain activation studies (Shulman et al., 2001). However, in this case the enhanced glucose uptake relative to oxidation would result in increased lactate production which would have been seen in the present study.

Alternatively, net glycogen synthesis could be occurring in which case there would be an equivalent increase in glucose uptake resulting in a lower OGI without an increase in lactate production. In the early preparative aspects of the PET studies of awake children, glycogen could have been depleted prior to the CMR_glucose_ measurement due to stress, sensory stimulation, or alerting, then re-synthesized during the assay interval. Evidence exists from animal models supporting enhanced glycogen breakdown under conditions of stress and increased arousal (Dienel and Cruz, 2016). In the present study, however, the children were anesthetized and glycogenolysis is not likely to contribute to lactate production above the low basal level.

### Metabolic studies in children underscore the difficulty and complexity of accurate, fully-quantitative determinations of non-oxidative metabolism of glucose with developmental age

**Figure 7:**
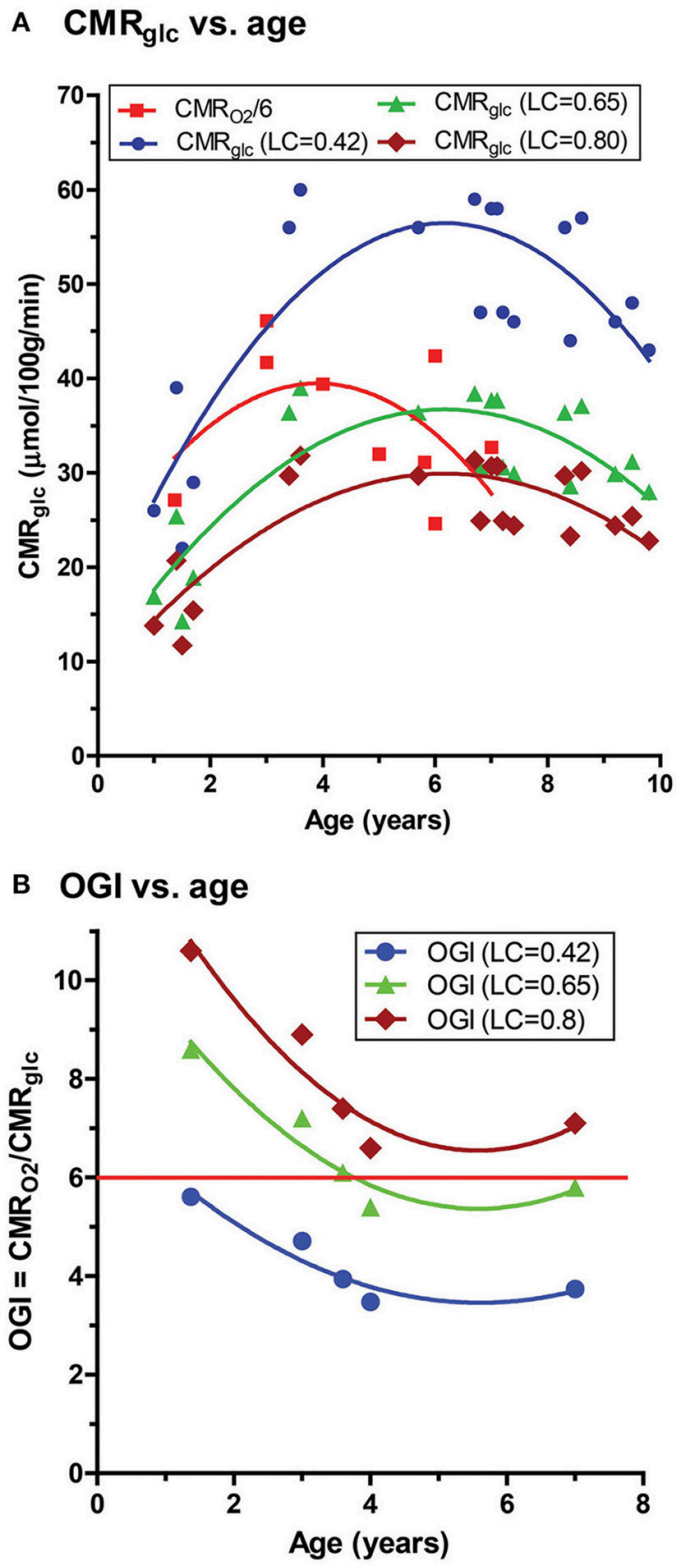
Calculated CMR_glucose_ and OGI as function of age in children. Values for children from age 1-10 years were taken from Supplementary Table 1 of Goyal et al. **(A)** Based on the ages of the subjects in the Goyal et al. (2014) and Chugani et al. (1987) all CMR_glucose_ data for this age group were assumed to be from the study of Chugani et al. who used a value of the lumped constant (LC) of 0.42 to calculate CMR_glucose_ in their [^18^F]FDG-PET studies (blue symbols and lines). For comparison, values at each age were recalculated using updated values for the LC, i.e., LC = 0.65 (Wu et al., 2003) (green) and LC = 0.80 (Wienhard, 2002; Hyder et al., 2016) (brown). To directly compare the different CMR_glucose_ data sets to the CMR_O2_ values reported by Goyal et al. each CMR_O2_ was converted to glucose equivalents (red squares) by dividing by 6 (which assumes all oxygen consumed is due to glucose oxidation–see text), with the caveat that correction for lactate fluxes and ketone body utilization that were not measured in these studies will alter these values (see Table 4). **(B)** OGI values tabulated by Goyal et al. were similarly corrected using LC = 0.65 or 0.8. The horizontal red line represents the theoretical maximum of 6.0 (see text). The solid curved lines are quadratic nonlinear regression lines calculated with GraphPad Prism 5.

Due to the invasive nature of methods for measuring brain glucose and oxygen consumption as well as concerns regarding radiation the number of brain metabolic studies in children is highly limited. The reports by Kennedy and Sokoloff (1957) and Mehta et al. (1977) (Table 4) were the only ones (to our knowledge) to measure CMR_O2_ in awake children. Kennedy and Sokoloff stated that there was no correlation of CMR_O2_ with age between 3 and 11 years, and dividing the Chugani values for CMR_glucose_ into the mean CMR_O2_ gives results (Table 4) similar to the OGI profile shown for LC = 0.65 in Figure 7B. In sharp contrast, use of the lower value for CMR_O2_ from N_2_O-anesthesized children resulted in much lower OGI values due to low CMR_O2_ in the anesthetized children (Table 4). In both cases, the OGIs do not reflect the true oxygen/substrate ratio because the contributions of lactate efflux and ketone body influx are not included. Notably, CBF in awake children (studies 1 and 2, Table 4), exceeded that in awake adults, whereas CBF in N_2_O-anesthetized children was lower than in awake adults and was 58% that of awake children in the same age range (studies 2, 6, and 7, Table 4).

Inspection of Figure 2A and Supplemental Table 1 of Goyal et al. indicates that most of the data for the 3–10-year-old children came from two studies: Kennedy and Sokoloff (1957) and Chugani et al. (1987). Kennedy and Sokoloff reported *whole-brain* CMR_O2_, whereas Chugani et al. reported *regional* CMR_glucose_ (also see Table 4) so the regional or global metabolic rates are not congruent, as required for an accurate OGI. Use of higher cerebral cortical values for CMR_glucose_ compared with lower whole-brain CMR_O2_ will artifactually inflate the magnitude of the CMR_O2_-CMR_glucose_ mismatch. Regional variations in CMR_glucose_ and CMR_O2_ and stability of regional OGI in normal resting adult brain (Hyder et al., 2016) support the conclusion that errors will be incurred by combined use of global and regional data to calculate OGI.

A caveat to the majority of studies looking at metabolic changes during development is that often sedation or anesthesia must be used to study young children. Of importance, N_2_O stimulates brain norepinephrine release in a time- and concentration-dependent manner, with 60% N_2_O increasing norepinephrine levels by about 3-fold at 50 min (Yoshida et al., 2001, 2010), and may influence brain glucose and glycogen metabolism and brain metabolite levels (Dienel and Cruz, 2016).

### Lumped constant and accurate CMR_glucose_ values

Calculation of OGI requires *accurate* and *absolute* values for both rates to obtain a valid molar ratio of oxygen to glucose utilization. However, as previously mentioned, the OGI does not have any information about the fate of the glucose carbon consumed in excess of oxygen. During development, there is growth and remodeling of brain structures such as synapses, and Goyal et al. emphasized this process as an explanation for excess glucose consumption (Goyal et al., 2014). However, the studies used in the meta-analysis did not measure fluxes of metabolic pathways, and the fate of glucose is unknown and remains speculative. The results of the present study demonstrate that lactate levels are far, far lower than expected in developing brain, ruling out glycolysis with lactate accumulation as a major contributor to a fall in OGI in children. Re-calculation of CMR_glucose_ and OGI with updated values for the LC strongly suggests that calculated CMR_glucose_ is not as accurate as required, causing errors in OGI. The OGI variability in Figure 7B is probably due, in part, to the reported CMR_O2_ values that have fewer data points than CMR_glucose_ within this age range. These data would be most accurate if CMR_O2_ and CMR_glucose_ were sequentially determined in the same brain regions of the same awake subjects, which is extremely difficult, if not impossible, to carry out in young children. In addition, determination of the net utilization of lactate, ketone bodies, and other potential substrates in blood and inclusion in the oxygen/substrate indicies is necessary to obtain accurate measures of brain metabolism during development.

Another caveat is that the LC may change with age and also depends on the model (Kuwabara et al., 1990). One component of the LC is the ratio of the distribution space of FDG to that for glucose. Conceivably, the distribution spaces may change during maturation as astrocytes, neurons, and oligodendrocytes increase transporters and metabolic enzymes, but the LC may be relatively stable because it is a ratio. The LC has been shown to be similar in fetal and neonatal sheep (Abrams et al., 1984) and studies in developing brain have assumed that the LC is constant during development (Kennedy et al., 1978, 1982; Kato et al., 1980; Nehlig et al., 1988). Re-calculation of CMR_glucose_ with updated values for the LC does not invalidate the age-dependent changes in CMR_glucose_ reported by Chugani et al. (1987). In fact our measured brain [Lac] values, while consistently low at all ages, do reach a maximum at ~5 years which is the maximum CMR_glucose_ reported by Chugani et al. (1987).

However, and most importantly, any departure from the true value of the LC for FDG and from the true absolute rate of CMR_glucose_ will invalidate the calculated OGI across all ages, not just in young children. The analyses presented in Figure 7 and Tables 3, **4** raise serious concerns about the accuracy of the OGI profiles in developing brain and aging brain reported by Goyal et al. because they did not take the use of different LC values and utilization of supplemental substrates into account in their meta-analysis (Goyal et al., 2014). Note that if ketone body oxidation were measured and included in the OGI calculations for the youngest children in Figure 7B, the values for OGI > 6 would be reduced to close to or below 6 (see Table 4). Furthermore, Goyal et al. divided CMR_O2_ by 6 to get glucose equivalents, which assumes no other substrates are oxidized. If ketones are consumed this calculation introduces an error into the comparison of CMR_glucose_ and CMR_O2_ in their Figure 2A because ketone bodies have different O_2_ - substrate stoichiometries than glucose (legend, Table 4). While recognizing this issue, the same calculation was used in Figure 7 in the present study so the data sets in their and our studies could be compared. Even if the LC = 0.42 and calculated CMR_glucose_ are appropriate and valid in the study by Chugani et al., regional CMR_O2_ was not measured in the same brain regions in the same subjects at the same time, seriously weakening conclusions related to the magnitude of aerobic glycolysis in children. Moreover, as we show in this study, the commensurate lactate levels do not match their predictions based on non-oxidative metabolism of glucose, and thus it is premature to conclude that 33% of the glucose consumed by 3-8-year old children is not metabolized via the tricarboxylic acid cycle to consume proportionate amounts of oxygen.

The brain glucose in children in the present study measured by the LC model analysis was in the range of about 1.7–2.2 mM (results not shown), ~2 times higher than anticipated in adults at a similar plasma glucose level based on ^13^C MRS measurements and ^1^H MRS measurements at higher fields using pulse sequences optimized for glucose detection (Gruetter et al., 1992, 1998; de Graaf et al., 2001; Shestov et al., 2011). From four studies in normal adults (Gruetter et al., 1992, 1998; de Graaf et al., 2001; Shestov et al., 2011), the brain/plasma ratio for glucose is about 0.2, and if this ratio is the same in children and we use the mean value for brain glucose for children aged 0.5–15 years old (under N_2_O) of 4.89 mM (Table 4), the expected brain glucose level would be 0.98 mM. Plasma glucose levels in adults in which the LC of 0.42 (Phelps et al., 1979; Huang et al., 1980) and 0.65 (Wu et al., 2003) were within the range 5.1–5.5 mM, with an anticipated brain concentration range of 1.0–1.1 mM. Due to limitations in the pulse sequence for measuring brain glucose in our study, the glucose values can be deceptive in that there can be poor accuracy but good precision (low Cramer Rao bounds, which in our study was in the range of 10–30% for glucose), and it is likely that the brain glucose concentrations in children are not accurate and are overestimated. Nevertheless, addressing this question in future studies is of importance for obtaining accurate CMR_glucose_ assays in children. Both brain and plasma glucose levels will impact the value of LC, with higher brain levels of glucose leading to somewhat lower LC values and correspondingly-higher calculated CMR_glucose_, as shown for [^14^C]deoxyglucose in adult animal studies (Schuier et al., 1990; Suda et al., 1990; Dienel et al., 1991). The relationships between the LC for FDG and brain and plasma glucose levels need to be determined in humans across age.

To summarize, the use of supplemental fuel and metabolic assays in different brain regions in different cohorts in which nutritional status was not matched will cause errors in calculated OGI. Higher metabolism of glucose via the pentose shunt in young children with Fru-6-P recycling and release of glucose carbon as CO_2_ is an important potential contributor to consumption of glucose in excess of oxygen. These issues must be evaluated quantitatively before the validity of the DG method is challenged. In this regard, regional CMR_glc_ reported by Chugani et al. (1987) for normal adult brain (0.2–0.27 μmol/g/min) with LC = 0.42 determined in adult brain in a separate cohort is similar to the whole brain CMR_glc_ (0.26 μmol/g/min) reported by Hyder et al. (2016) using FDT-PET and LC = 0.8 determined in the same subjects, and adult whole brain CMR_glc_ reported by Madsen et al. (1995) (0.23 μmol/g/min), calculated from measurements of cerebral blood flow and arteriovenous differences. As discussed above, the updated values for the LC can arise for various technical reasons, and their use in the present study illustrates the effects of uncertainties in the true value of the LC in young children on the temporal profile of OGI. Changing the value of the LC increases OGI, which would be too high when supplemental fuels are used but not taken into account.

### Limitations of the study

The ability to measure resting lactate by ^1^H MRS has been criticized based on its low levels and contamination from brain macromolecules and lipids from the skull (arising due to incomplete volume localization). However, based on examination of spectra (Figure 3) the outer volume lipid contamination was minimal. Furthermore, excellent Bo homogeneity was achieved so that the lactate methyl group doublet at 1.33 ppm was well-resolved from the broad macromolecule peak at 1.3 ppm underlying lactate which would minimize the possibility of lactate spectral intensity being assigned to the macromolecule peak in the fitting process. Furthermore, if all the resonance intensity at 1.3 ppm were due to lactate, its concentration would be at most ~1 mM (Figure 3), which is still well-below what would be needed to explain the reported OGI.

The measured values of lactate as a function of age could potentially be influenced by changes in water and metabolite relaxation as a result of changes in water content and the cellular microstructural environment. Due to the challenges of studying young children there are only a limited number of studies looking at T2 relaxation. However, an extensive study at 1.5 T by Leppert and coworkers found that the T2 of water in gray and white matter rapidly decreased after birth reaching a constant value between 10 months and 5 years (Leppert et al., 2009) which encompasses the age range of children in our study (and at values of T2 similar to those measured in adults). Although there are no studies of lactate T2 changes with age it is unlikely that it would be more sensitive than H_2_O which undergoes extensive exchange with macromolecules and other compounds. Furthermore, the T2 of lactate in adults at 3T is ~200 msec (Cady et al., 1996) so that even if it is higher in children there would be little impact on the relative quantitation of lactate for a TE of 32 msec. Although the macromolecule T2 is on the order of the TE (Behar et al., 1994) there is no evidence of a change in the linewidth or relative intensity of the 1.3 ppm macromolecule peak with age so that the effectiveness of the LC model in separating lactate from macromolecules would not be age dependent.

A possible confound of the present study is that the children were studied under sevoflurane or propofol anesthesia (Jacob et al., 2012). However a recent ^1^H MRS study has shown that lactate is, in fact, elevated in mice anesthetized with volatile halogenated anesthetics (Boretius et al., 2013), suggesting that the awake [Lac]_B_ values may be lower. Thus, the measured [Lac]_B_ in the current study, and therefore the CMR_Lac_ determined from it, should be considered as maximal estimates. Furthermore, our results are similar to a 3T study recently published using ^1^H MRS to measure brain [Lac] in a smaller group of neonates and children (Tomiyasu et al., 2016). Another possible confound could be attributed to the variable anatomical voxel location for the ^1^HMRS spectra which was not consistent across subjects. We therefore acknowledge that there might have been minor variance in the data due to region dependent differences in metabolite levels.

Dienel and colleagues (Ball et al., 2010) have shown that during activation up to 25% of lactate can diffuse out of regions where it is produced by mechanisms independent of blood flow and, therefore, lead to an underestimate of regional brain lactate efflux from lactate levels alone. However, given the non-activated (anesthetized) conditions such as the present study it is unlikely that these mechanisms would lead to a significant underestimate since the entire cerebral cortex of anesthetized children is at a similar level of activity and presumably lactate production. Future studies using MRS imaging could further address the issue of lactate concentration heterogeneity.

Metabolic studies in children are particularly difficult to interpret because the duration of fasting influences plasma ketone body levels (the longer the fast, the higher the plasma ketone levels), brain ketone body utilization is linearly related to plasma level, and younger children take up ketones better than older children or adults at the same plasma level. Notably, Kennedy and Sokoloff reported no age-dependence of CMR_O2_ in their awake 3–11-year-old cohort, and Settergren et al. (1980) also reported no age correlation with CBF, CMR_O2_, CMR_glucose_, CMR_lactate_, and CMR_ketones_, in 1–15-year-old N_2_O-anesthetized children (study 6, Table 4) contrasting the age-dependence of CMR_glucose_ in the awake children in the Chugani study (study 8). To summarize, the contributions of N_2_O, alerting, stress, fear, and other factors on these metabolic differences remain to be evaluated, underscoring the need for caution in interpreting results of a meta-analysis in which oxygen and total substrate utilization were not measured in the same subjects and brain regions at the same time. Many factors complicate interpretation of metabolic rates and OGI in children.

## Conclusions

Using ^1^H MRS we found that [Lac] in cerebral cortex of young children was very low, and that the maximal calculated efflux of lactate cannot explain the mismatch between CMR_glucose_ and CMR_O2_ previously reported, in agreement with results of the previous metabolic studies in 3-15-year-old children summarized in Table 4. Depending on plasma lactate levels it is possible that there was a net small influx of lactate. The results of this study rule out an increase in glycolytic rate and accumulation and release of lactate as a primary cause of elevated non-oxidative metabolism of glucose reported in young children. Possible explanations for utilization of the “missing” glucose in excess of oxygen are carbon loss as CO_2_ via the pentose phosphate pathway, use of carbon for nucleotide synthesis, protein synthesis, lipid synthesis, and glycogen turnover with lactate efflux. However, the most significant sources of disagreement are likely the (i) validity of assumptions made in PET studies regarding the true value of the LC, which we believe requires that the present understanding of how OGI and CMR_glucose_ change with age be reexamined, (ii) use of fuel in addition to glucose, and (iii) assays of CMR_glucose_ and CMR_O2_ in different brain regions of different subjects. To summarize, enhanced non-oxidative metabolism of glucose during brain maturation is a complex phenomenon to which many metabolic pathways, fuel sources, and technical issues have a strong influence. Brain developmental progress certainly plays a role in metabolic changes with age, but their quantitative contributions remain to be established. In spite of these interpretive limitations, ^1^H MRS provides a potentially valuable new biomarker for assessing non-oxidative metabolism of glucose in infants and children and studying its relationship to brain development in health and disease.

## Ethics statement

The study uses de-identified data from a previous published human IRB approved study (Jacob et al., 2012). The original study by Jacob et al. (2012) was carried out in accordance with the recommendations of Federal Regulations Department of Health and Human Services (DHHS)/Office for Human Research Protections (OHRP), USA. The protocol was approved by the IRB committee at Stony Brook University (CORIHS). All parents of the children gave written informed consent in accordance with the Declaration of Helsinki.

## Author contributions

HB and DR conceived the study. HB, DR, and GD performed the analyses; GD conceived the revisiting of the lumped constant for FDG conversion and calculation of OGI. HB, ZJ, and RM designed the original ^1^HMRs experiments. HL performed the LCModel analysis on ^1^H MRS spectra and the volumetric analysis. HB, DR, and GD wrote the paper. AG and FH posed scientific questions, read and revised the manuscript. All authors edited and reviewed the paper.

### Conflict of interest statement

The authors declare that the research was conducted in the absence of any commercial or financial relationships that could be construed as a potential conflict of interest.
